# Democratizing education: Open schooling engaged the less privileged in environmental sciences

**DOI:** 10.1371/journal.pone.0266655

**Published:** 2022-04-08

**Authors:** Hilde Karine Wam, Agata Goździk, Paul Eric Aspholm, Tomasz Juńczyk

**Affiliations:** 1 Division of Forest and Forest Resources, NIBIO, Ås, Norway; 2 Institute of Geophysics, Polish Academy of Sciences, Warszawa, Poland; 3 Adam Mickiewicz University, Faculty of Educational Studies, Department of Methodology of Educational Sciences, Poznań, Poland; University of Macau, MACAO

## Abstract

Democratizing learning is essential for environmental sustainability. Less privileged areas are crucial in this regard. Informal education has great such potential, but often fails to reach the less privileged, and to document learning. With the objective to identify and counter these issues, we here report on EDU-ARCTIC, an informal open schooling course in environmental science, aimed at European teachers with teenage pupils. Of the 1,181 teachers who enrolled, 73% were females and 43% were from less privileged nations (according to UN Human Development Index). This is a higher share of less privileged (females) than is the case for the general population of Europe. Teachers from less privileged nations also participated in more project activities than did those from more privileged nations, apart from in urban areas. For the project period, the teachers reported a significant increase in all the three categories of aspired learning outcomes for their pupils. We conclude that courses like ours can increase teenagers’ literacy and engagement in science and environmental issues, not the least in less privileged areas. Deliberate efforts are required to reach these target groups, who may be less inclined to join on their own.

## 1. Introduction

The provisioning of open online courses continues to grow [1, 2], and now ostensibly engages three-digit millions of learners [1]. Its foremost advocacy has been the potential to democratize learning–by easing academic access for less privileged groups [3, 4]. This gives it fertile grounds, because equal access to education for all people is a committed goal of major international signatories. It is, for example, considered a critical tool to facilitate sustainable citizenship [5 - target 4.7, 6], which we here define as any personal action that either lessens one’s own socio-ecological footprint [sensu 7], or promotes such actions by other citizens for the common good.

Surprisingly, only a fraction of the academic open online courses target environmental sciences. More than 900 universities currently offer massive open online courses (MOOCs) for a total of 50 different degrees [1]. Less than 10% of these MOOCs have natural science as their main topic, and of that fraction, most are not environmental, but basic like pure physics or chemistry. While sustainable citizenship may be touched upon in other academic fields, we believe knowledge and skills from the transdisciplinary environmental science (formally considered natural science) is crucial to develop the personally critical mind-set [sensu 8] that is needed for sustainable citizenship. This may be more central in non-formal online courses, but for these there are no global statistics.

Despite the potential, open online courses have been strongly criticized for failing to democratize education, especially the mass-oriented courses. These appear to have drawn learners mainly from the more privileged nations and social groups [e.g., 9, 10]. Regarding nationality, language barriers likely are central, because the majority of MOOCs are given only in English. Regarding social groups, we would expect, for example, rural people to be particularly interested in online learning, because they generally have less optimal conditions for education [e.g., 11, 12, 13]. However, in a study with more than 40 000 online learners, geographical isolation from educational institutions was the *least* frequent motivation for having sought the online courses [14]. This possibly reflects a cultural-geographical bias in societal importance of education [e.g., 15]. For example, in Romania approximately 40% of pupils (primary to university) are from rural regions [16]. However, there is a greater drop-out of rural pupils, so by the time they reach university only around 10% are from rural regions. If the less privileged are less inclined to seek education by themselves, perhaps the most important failure to engage them for online courses is failure to reach out to them. We hereafter refer to this as **Challenge 1**, one of two challenges that we aimed to meet in this study.

Another and most important criticism of open online courses is lack of monitoring the students’ learning. There appears to be a general lack of emphasize on this [17, 18], despite so many of the courses being offered by renowned universities. While a given learning outcome can be achieved through many didactical approaches [and technologies, 19], any new education scheme needs to document its effect relative to its goals. Discouragingly, in Hollands and Tirthali (18) only 20% of 83 interviewees involved in the provision of open online courses raised ‘improved education outcomes’ as one of their goals. On a positive note, the peer-review literature addressing this lack is slowly catching up [see 20, 21]. The monitoring of learning comprised **Challenge 2** in our study.

**Fig 1:**
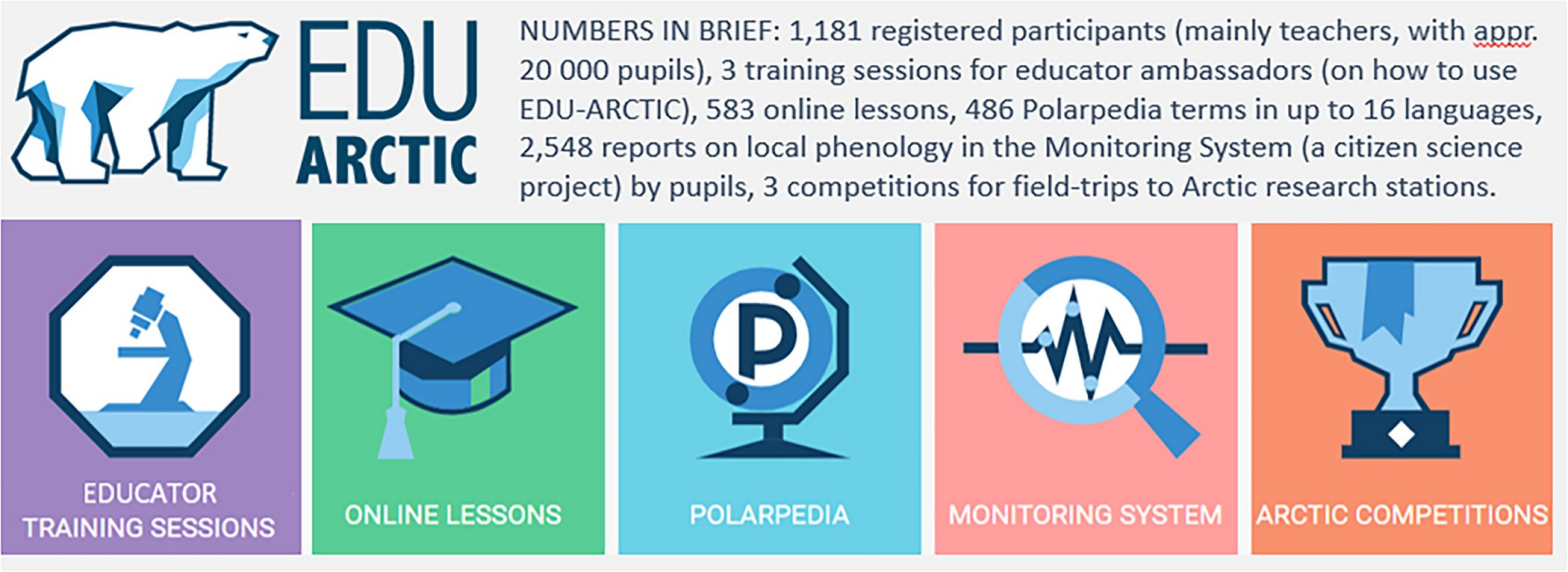
Overview of learning components in the EDU-ARCTIC open schooling course (2016–2019). All learning resources were freely available to anyone registering on the portal, at no cost, but specifically targeted European teachers with their students 13–20 years of age.

In this paper, we report on teacher-evaluation of pupils’ learning outcomes in a semi-massive and multi-linguistic open online course (“EDU-ARCTIC”) in environmental science, aimed at European teachers with their pupils 13–20 years of age (Fig 1). Because the project combined online and place-based activities, we also refer to it as ‘open schooling’ rather than only an ‘open online course’. We had *a priori* ambitions to counteract the challenges outlined above (**Challenge 1–2**). In brief, we pursued to reach the less privileged by investing more heavily in our recruitment of teachers from rural than urban, and from Eastern than Western Europe. We monitored and evaluated learning through structured online surveys for the teachers, who evaluated the learning outcomes collectively for the pupils in their class. This paper thus is a scholarly case study of how science-based open schooling affected young people’s literacy and engagement in environmental issues, their interest in pursuing a STEM career, and whether this was influenced by socio-geographical aspects of their school.

## 2. Materials and methods

### 2.1. Pedagogical approach

We followed a pedagogy that aim to engage curiosity, personal conscience and critical thinking rather than to indoctrinate about right and wrong [8]. This approach stemmed from the notion that sustainable citizenship springs out of a *caring for* the common good [*sensu* 22]. In line with this, we used traditional lecturing from teacher to pupil (webinars) only as part of the parcel. A flipped learning setting is likely to better create engagement and self-efficacy, as the pupils cannot only receive, but also have to seek out and interact with the learning materials on their own [2]. We strived to make even the lectures as interactive as possible, inviting the teachers and especially the pupils to speak aloud and to do simple exercises. We also offered other interactive learning tools, and encouraged the teachers to use these in a preparatory manner with their pupils. The teachers thereby had a variety of ways to blend EDU-ARCTIC into traditional in-class didactics (the webinars) and a flipped learning environment (the online learning tools such as Polarpedia, quizzes etc.). As there were no obligatory activities, it was completely up to the teacher how they organized this.

### 2.2. Organization and content of the course

EDU-ARCTIC was created as a singular and free-standing research and innovation project (2016–2019), and jointly organized by six research institutions or educational SMEs from five countries in Europe. The scientific contents of the project was created and presented by science-literate staff (academic researchers and educators), and put in a pedagogical framework in close collaboration with staff having longstanding teaching experience. The topics covered did not follow specific school curricula, but instead we broadly covered ongoing environmental issues, the importance of science and how it is to work as a scientist.

All learning modules (Fig 1) were freely available to any interested party who registered on the project portal (https://edu-arctic.eu/), and most were openly available even without registration. There were no fees involved, and when attending place-based activities, the participants had their travel, food and hotel expenses covered by the project. We had a monitored online forum for the participating teachers, where they could engage with each other or consult project staff. We provided continuous teacher support (e-mail, forum, and phone) operated during normal working hours.

The interactive webinars (online lessons) were live-streamed, each for a duration of 30–50 minutes. We streamed from a variety of places, some exotic, such as polar research stations at Svalbard and in the northernmost of Norway. Webinars were recorded and later made public on our YouTube channel (https://www.youtube.com/c/EDUARCTIC/about). A total of 583 webinars were offered by 40 different researchers/educators from 2017-01-12 until 2019-06-17, of which 244 were unique topics (S1 Table in S1 File). Some topics were given several times or in several languages. Of the webinars offered, 560 were attended by at least one participant. There were on average 5.4 webinars offered per week (excluding weekends, and Jul-Aug), and these were attended by an average of 8.8 ± SD 6.2 teachers with an estimated 16 attending pupils per class, for a total of 5,112 “teacher-hours” and about 79,000 “pupil-hours”. The teachers had to sign up for webinars, as there was a technical space limit of 23 teachers with their class of pupils per webinar. Many webinars were fully booked, with waiting lists, but there were quite a lot of no-shows, and only 5 webinars were fully attended.

Directly accompanying the webinars, we continuously updated a course encyclopedia of relevant terms. This included easy-to-understand explanations, illustrations and animations designed to inspire the pupils to seek further resources for more in-depth learning. For several webinar topics we also created learning materials in the form of worksheets, quizzes and online games. These were usually available to the teachers prior to the webinars. We offered webinars in up to 10 languages and learning materials in up to 16 languages upon demand, provided we had linguistically qualified staff.

Environmental issues are highly value-loaded [23], and in line with the natural science tradition, we aspired to present facts as value-neutral and as balanced as possible (i.e. presenting all relevant facts, not biasing by omission). In addition to the scientific literacy, we also tried to show examples of how science is part of everyday life, and thus integral to sustainable citizenship. We also had two learning-by-doing modules specifically designed to engage pupils to become more participatory citizens [24], with emphasize on stimulating pupils to ‘ask the good questions’ [25]. ‘The Monitoring System’ was a citizen science portal, where pupils reported meteorological observations and local phenology like the first flowering of a plant [26]. A total of 2,548 observations were submitted, time-stamped and geo-localized, and made available for all teachers to use in their own school projects. ‘The Arctic Competition’ (one in each of three years) let the pupils work singularly or in teams throughout the school year with their own idea for a science project under the guidance of their teacher, with help from project staff available on demand. A total of 277 ideas were submitted from 23 countries in the form of essays or media. Through several evaluation stages, a few finalists were granted a multi-day stay on a polar research station, to test out their idea in collaboration with researchers.

### 2.3. Study area (Challenge 1: Reaching the less privileged)

The consortium was compiled partly to have partners representing opposite localizations within Europe along the rural-urban divide as well as on the UN Human Development Index. From most to least rural: Pasvik in Norway (Norwegian institute of bioeconomy research, NIBIO), Akureyri on Iceland (Arctic Portal), Torshavn on the Faroese Islands (Faroese Islands Nature Investigations, FINI), Poznań in Poland (American Systems Sp. z.o.o.), Versailles in France (University of Versailles Saint-Quentin, UVSQ) and Warsaw in Poland (Institute of Geophysics Polish Academy of Sciences, IGF-PAN). This facilitated local knowledge that we used to reach out to rural and/or socio-economically less privileged communities, essentially in their native language.

We applied a range of tools to reach our target groups, most prominently social media, in-school visits and articles in educational magazines, but also phone calls, e-mails, newsletters, newspapers and various educational events (see also section 4.1). We assumed that online outreach would sufficiently reach urban teachers, while rural teachers needed a more personal contact. In our school visits, we therefore prioritized the more rural communities. We motivated and monitored activity of teachers by assigning each with an accumulating activity score (“Edu-Game”), where various activities gave different points (S1 Table in S1 File). We continuously updated a public list of top scorers (first name and nation) in our digital media. Top scorers received special awards like exclusive online lessons for their class and diplomas. Notably, we also gave scores for filling in the evaluation surveys (section 2.4).

In this study, we considered ‘less privileged’ at two levels: (1) By the country’s value on the United Nations Human Development Index (adjusted for inequity, IHDI) [27], which is based largely on education indices, and therefore should be particularly applicable for our purpose. We defined as ‘less privileged’ countries that were below the median for Europe = 0.815. (2) By the population density of the area where the participating teacher’s school was located. We here defined rural areas as ‘less privileged’, because rural teachers are likely to have fewer place-based learning opportunities, such as teacher continued education courses and informal learning through colleagues [28]. We defined urban, suburban and rural areas following the Eurostat Regional Yearbook [29]: ‘urban’ >1,500 people per km^2^ (called urban centres in Eurostat), ‘suburban’ 500–1,499 people/km^2^ (called urban clusters in Eurostat), and ‘rural’ the remaining. For 97 locations we did not obtain reliable densities, and used instead the national definition of whether the area was a city (urban), a town (suburban) or a village (rural). In 18 very rural cases, even this information was unavailable, and then we used satellite images to verify that the area had dominance of agriculture land and an absence of dense buildings. There were 5 teachers who we could not assign to any category due to erroneous spelling of their school location.

### 2.4. Data collection (Challenge 2: Monitoring of learning)

The specific goals of learning outcomes for the pupils were:

1. *Enhanced knowledge about nature in polar areas and its global role in environmental issues*.
2. *Enhanced understanding of research and scientific language in environmental sciences*.
3. *Familiarization with scientific career opportunities*, *and increased interest in pursuing such*.

During the project, we used a mixed method approach of quantitative surveys and focus group interviews to collect data on learning in our project, in order to gain the most insights to improve our course [30]. The latter may better facilitate, for example, spontaneous feedback, while the former provides for hard data and statistical testing. Here we report on the quantitative data. We carried out the surveys online, which creates a sense of anonymity and the opportunity to participate at a convenient time, allowing more deeply reflected responses [31]. We distributed the surveys to teachers by e-mail, with automated reminders. We present our two surveys pertaining directly to learning outcomes; the ‘pre-survey EDU-ARCTIC skills assessment’ and the ‘post-survey EDU-ARCTIC skills assessment’. We had additional surveys to obtain feedback for improving the project while it was running [see 32, 33]. These were valuable for implementation of the project, but are less relevant here. The teachers evaluated collectively all pupils in their class, giving an average score split for boys and girls. The two surveys asked the same set of questions (S1 Table in S1 File, response data in S1 Dataset), so that the pre-survey indicates the pupils’ literacy and engagement relevant for the learning goals *prior to participating* in the project, while the post-survey indicates the same parameters *afterwards*. The change from pre-survey to post-survey indicates learning outcomes of EDU-ARCTIC.

We opted to have the teachers evaluate the learning outcomes of their own pupils. Our approach is based on the crucial recognition that learning is driven by not only cognitive functions, but also emotional and somatic ones [34]. While humans gradually develop towards more wisdom with age [35], the processes are not always linear and certainly not uniform [36]. The teachers should therefore be better evaluators of their pupils’ performance than are the pupils themselves, and certainly better than a purely cognitive test or a qualitative evaluation conducted by external educators unfamiliar with the individual pupil. The teachers are highly skilled at observing the learning of pupils and comparatively so to the pupil’s peers. Ideally we wanted evaluations for individual pupils, but we anticipated that this would be too much to ask of the teachers, who generally are short of time and loaded with tasks. Instead of running the risk of receiving too few survey respondents, we asked teachers to do the collective evaluations (as outlined above). Potential bias from our choice of evaluation method is further addressed in our discussion (section 4.2).

### 2.5. Statistical analyses

We analyzed all data quantitatively in the open source software R, version 3.5.1 [37]. All data were included in the relevant analyses, and no outliers were omitted. We generally checked assumptions of statistical models (all linear) by visual inspection of plots of residual versus fitted values [38], specifically scale-location plots (using standardized residuals). In most cases, our explanatory variables were categorical (or binary). For all models presented in this paper, the plots showed a near flat fitted: residual relation (same variance across predictor values, i.e. no heteroscedasticity).

We tested if gender, UN Human Development Index (IHDI) and rurality were related to (1) the likelihood of registered teachers becoming active, using logistic regression, ‘glm’ in R, with family binomial, link ’logit’ and response variable yes = 1 and no = 0, (2) their overall activity score, and (3) their activity score for specific types of activities like place-based versus online. For (2) and (3) we used linear regression (‘lm’ in R). The activity score was skewed towards lower scores, with a tail of some few very active teachers. We exploratory ran our models with the activity score variously transformed. However, we obtained the same results and significance, so in the paper we present the tests with non-transformed data, to facilitate a more direct interpretation of the parameter coefficients. For the IHDI index we tested both the numerical value itself, and a corresponding categorical variable we called ‘less privileged’ with levels yes = 1 and no = 0. For the numerical IHDI we applied simple Pearson correlations to alternatively indicate the extent of its relationship to the response variables.

We tested reported learning outcomes by comparing the results of the pre-survey and the post-survey. The response variables were in the form of tick-boxes with Likert scales 1–4 or 1–5, with explanatory text (where 4 or 5 indicated the highest level of skill, knowledge, use, interest etc.). The teachers filled in the number of pupils (girls and boys separately) in their class that they considered to belong to each level on the Likert scale. From our previous reports from the project [33], we knew that there were no strong differences in the teachers’ evaluations of girls and boys (generally the skill levels were reported to be higher for girls, but the relative increase in the scores from the pre- to the post-survey were approximately the same for the genders). The pupil genders were therefore merged in this study.

For each question in each survey, we calculated the mean score for the teacher’s class weighed by the number of pupils in each Likert category level (number of pupils in category level*value of category level (e.g., 1, 2, 3), divided by the sum of pupils across all the category levels), and treated this statistic as the numerical response variable in our subsequent analyses. The numerical response values were adequately normally distributed (somewhat skewed towards higher scales, but still clearly bell-shaped), so we applied no transformations of these data. The validity of this choice was also confirmed by lack of patterns in the subsequent residuals plots. Because not all teachers filled out both forms, we mainly present the data pooled across teachers, because we were also interested in aspects of only the pre-survey. However, to investigate eventual bias from who filled in which survey (see discussion section 4.2), we also ran the analyses with paired data for the respondents (N = 65) that had filled in both the pre- and post-surveys. To make it easier for the reader, we present these latter analyses only for the teacher’s average scores across all survey questions. The bias in question would have applied equally to any question.

We applied linear models (‘lm’ in R) to test for differences in mean response values between the pre-survey and the post-survey (‘pre’ and ‘post’ used as categorical explanatory variables). We likewise tested for the influence of IHDI listing on levels of evaluation scores in the pre-survey, to see if this differed between countries more or less privileged. We did not test the same for gender as we believed sample sizes were insufficient for males (N = 60 males in the pre-survey and 17 in the post-survey). We neither tested this for rurality, because the survey data were only linked to country, not school localization.

For most of the questions, the teacher had the opportunity to select ‘I have no opinion’. Of the total 8,502 responses (questions*respondents) asked for, teacher selected this option only 3% of the times. For this reason, the underlying pupil sample sizes for separate questions may be slightly lower than by the indicated general number of pupils in the pre- and post-surveys. There were eight teachers in the post-survey who were not in the pre-survey. There were also two teachers who filled in the surveys more than once, because they used the course to teach more than one class. We opted to keep all surveys from these two respondents.

### 2.6 Ethics

In order to ensure the highest standard of implementation as far as ethical issues are concerned, the Consortium requested a statement of opinion from the Committee on ethics in scientific research of the Institute of Geophysics PAS’ Scientific Council. The Committee was provided with information on the project, its objectives, activities, in particular issues related to the participation of adolescents in project activities, as well as personal data protection. Moreover, information on the informed consent procedures, templates of informed consent forms, information sheets and clarification of the children’s assent procedure were presented in detail. The Committee stated that EDU-ARCTIC is compliant with recognized ethical standards and provisions of the European Charter for Researchers and issued a positive opinion on the project. All registered participants (teachers) were informed on the purpose of collection of data and feedback surveys, and sent a written online consent form to be completed during the process of registration. Participation in all surveys was voluntary.

## 3. Results

### 3.1. Challenge 1: Reaching the less privileged

**Fig 2:**
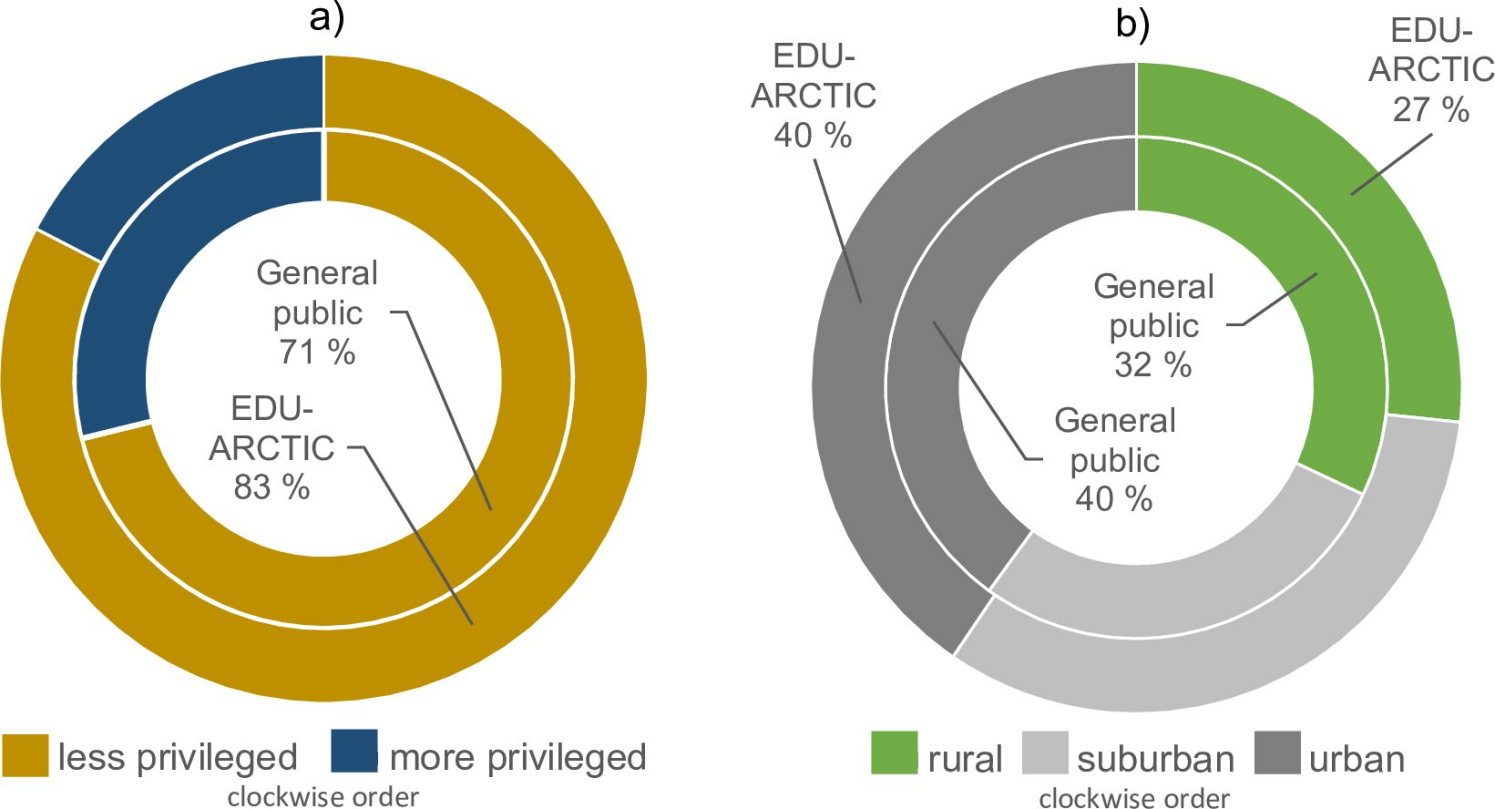
Proportion of less privileged groups among 1,176 teachers participating in the open online course EDU-ARCTIC (2016–2019) and among the general European population, according to **a)** country’s value on the United Nations in-equality adjusted Human Development Index (IHDI), from which we defined less privileged as below median for Europe (= 0.815). **b)** rurality of the teacher’s school, where we defined rural areas as <300 people/km^2^, suburban as 300–1,500 people/km^2^ and urban as >1,500 people/km^2^ (*sensu* the Eurostat Regional Yearbook). Both a) and b) are based on individual teachers (some schools had >1 teachers participating).

In total, 1,181 unique teachers registered on the EDU-ARCTIC portal (from 2016-12-14 until project activities officially closed 2019-06-30), omitting test accounts and duplicates. There was a high share of females (73%). The teachers came from 60 countries. In terms of individuals, 91% came from Europe. Poland was the dominating country with 35% of the teachers, thereafter Romania (10%), Albania (9%) and the Faroe Islands (7%). The proportion of teachers from less privileged countries were 12 percent-points higher than is the case for the general population of Europe (Fig 2A). The teachers came from 586 different places (cities, towns, villages). The median population density of these places was 52 people/km^2^ (rural), 660 people/km^2^ (suburban) and 2,800 people/km^2^ (urban). The proportions of rural, suburban and urban teachers followed that of the general population of Europe (Fig 2B). Notably, a *lower* share of the rural teachers were from *less* privileged nations (69%) compared to the urban teachers (91%). Particularly rural *males* (N = 86), who less often than females were from less privileged nations (log odds β = -0.72±0.25, z = -2.8, p = 0.005).

**Fig 3:**
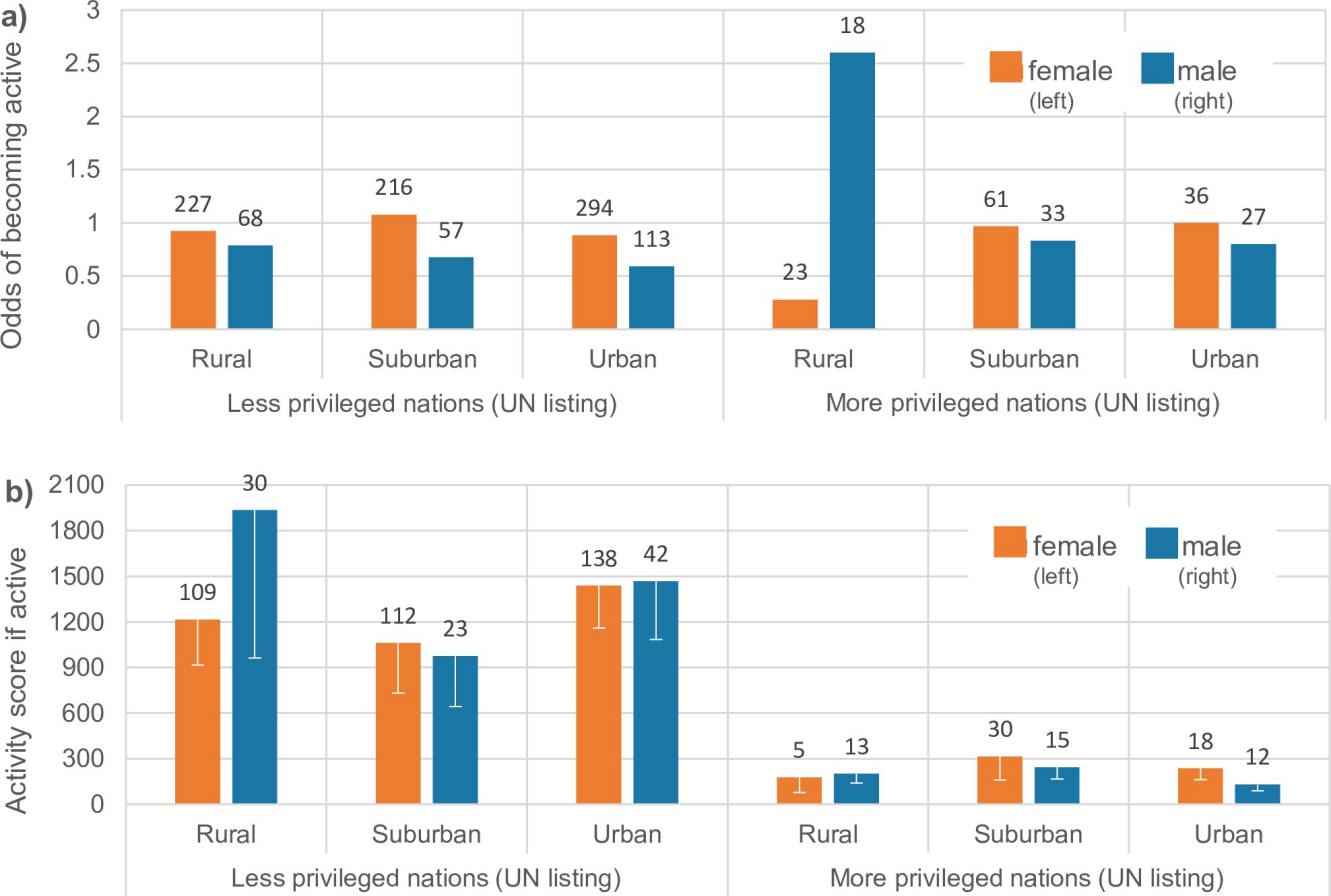
Activity among sociodemographic groups of teachers participating in the open online course EDU-ARCTIC (2016–2019). Less privileged = nations below median United Nations Human Development Index for Europe (IHDI = 0.815). Rural (<300 people/km^2^), suburban (300–1,500) and urban (>1,500) *sensu* the Eurostat Regional Yearbook. Numbers above bars are sample sizes (numbers of teachers in that sociodemographic group). **a)** Likelihood (odds) of becoming active among all 1,176 teachers registering for the course and who stated their school localization. The odds should be read relatively to each other, so that a higher bar means this demographic group was more likely to become active, and not just register for the course. **b)** Activity score are mean ± 1 SE for the 547 teachers (with localization) who did become active. Median score across groups was 200. There were no significant differences among sociodemographic groups in the activity score.

Among the teachers who registered as participants on the project portal, 55% never attended any score-eligible activity (i.e. they may have been completely inactive, or they may have browsed project material freely available on the portal), while 45% were active in at least one such activity. Note that only active teachers participated in the learning outcome surveys. There was a lower likelihood to become active for males than females (log odds β = -4.4±1.5, z = -3.0, p = 0.003), albeit less so in more privileged nations (gender*IHDI interaction z = 2.8, p = 0.005). The latter was due mainly to males in rural higher IHDI areas (Fig 3A). If active, both genders had substantially lower activity scores if they were from more privileged nations than if they were from less privileged nations (t = -4.1, df = 556, p≤0.001) (Fig 3B). The activity score showed strong negative correlation with the IHDI value (Pearson’s = -17.1, df = 556, p<0.001). For rural teachers, the score was also positively correlated with the population density of their localization (Pearson’s = 32.9, df = 146, p<0.001). This means that teachers in the most scarcely populated areas (of nations with higher IHDI) were the least active.

The active teachers were on average active over a period of 355 ± SD 320 (median 291 days, ranging from 1 to 1,187). The maximum number of days one could be active was 930 (from registrations first opened on the portal 2016-12-14 until the project activities officially closed 2019-06-30). Only 15 teachers cancelled their registration before the project ended, so the difference in days active stems from registration dates, not cancellation dates. About half the teachers were active <100 days (57%), while 15% were active >500 days. Teachers from less privileged nations were active for longer than teachers from more privileged nations (t = 3.0, df = 556, p = 0.003).

**Fig 4:**
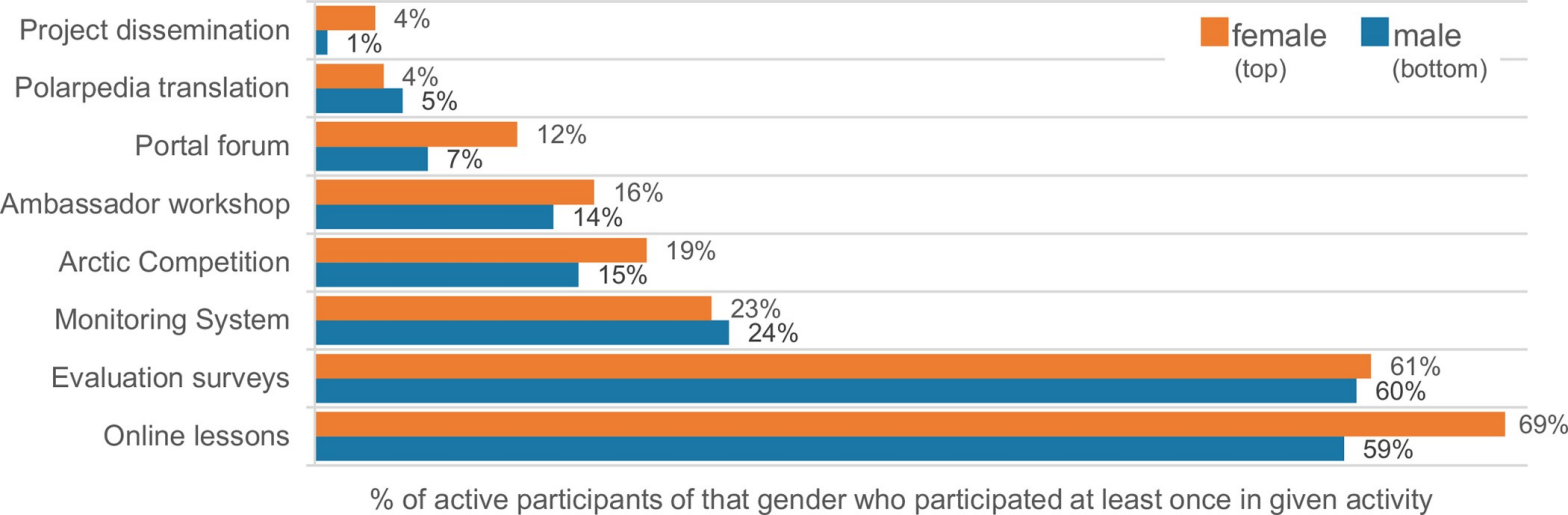
Participation in various activities of male and female teachers in the open online course EDU-ARCTIC (2016–2019). Percentages are based on active teachers, and calculated per gender (N = 412 females, 135 males).

We had both online and place-based activities. Almost all active teachers (92%) participated in at least one kind of online activity, while 41% were active in at least one kind of place-based activity. We found no strong influence of gender, IHDI listing or rurality on the likelihood of participating in a place-based activity. On average, teachers were active in 2 ± SD 1.4 out of 8 available types of activities (median 1) (Fig 4). Males tended to be active in fewer types of activities than females (t = -1.6, df = 556, p = 0.112). The mean number of enrolments per active teacher for online lessons (the core of the course) was 9 ± SD 29.8 lessons, while the median was only 1 (range 0–303). In other words, some teachers were highly active for lessons, while the majority were far less active. Summed across all activity types, 77% of the teachers were recorded as active less than 10 times, and 11% more than 50 times (participants, both teachers and pupils, also used course resources without being recorded as active, such as the Polarpedia).

### 3.2. Challenge 2: Monitoring of learning

Of the active teachers, 47% (N = 255) filled out the pre-survey, and 13% (N = 72) filled out the post-survey. Their number of pupils was negatively correlated with their IHDI value (t = -2.7, p≤0.000), albeit the variation was high (Pearson’s only -0.15). Teachers in the pre-survey altogether reported to teach 12,469 girls and 12,929 boys (not necessarily all using EDU-ARCTIC), while those in the post-survey reported to teach 1,776 girls and 1,652 boys. There were bias in which teachers completed which surveys (see 4.2 discussion). For example, the activity score when the project ended was substantially higher for teachers in the post-survey (median 2,099) than for teachers in the pre-survey (median 540) (t = 4.5, p≤0.001). Also, teachers in the post-survey were almost all from less privileged nations (N = 69 out of 72). The gender ratio, however, was not biased, and followed that of all registered teachers (76% females).

**Fig 5:**
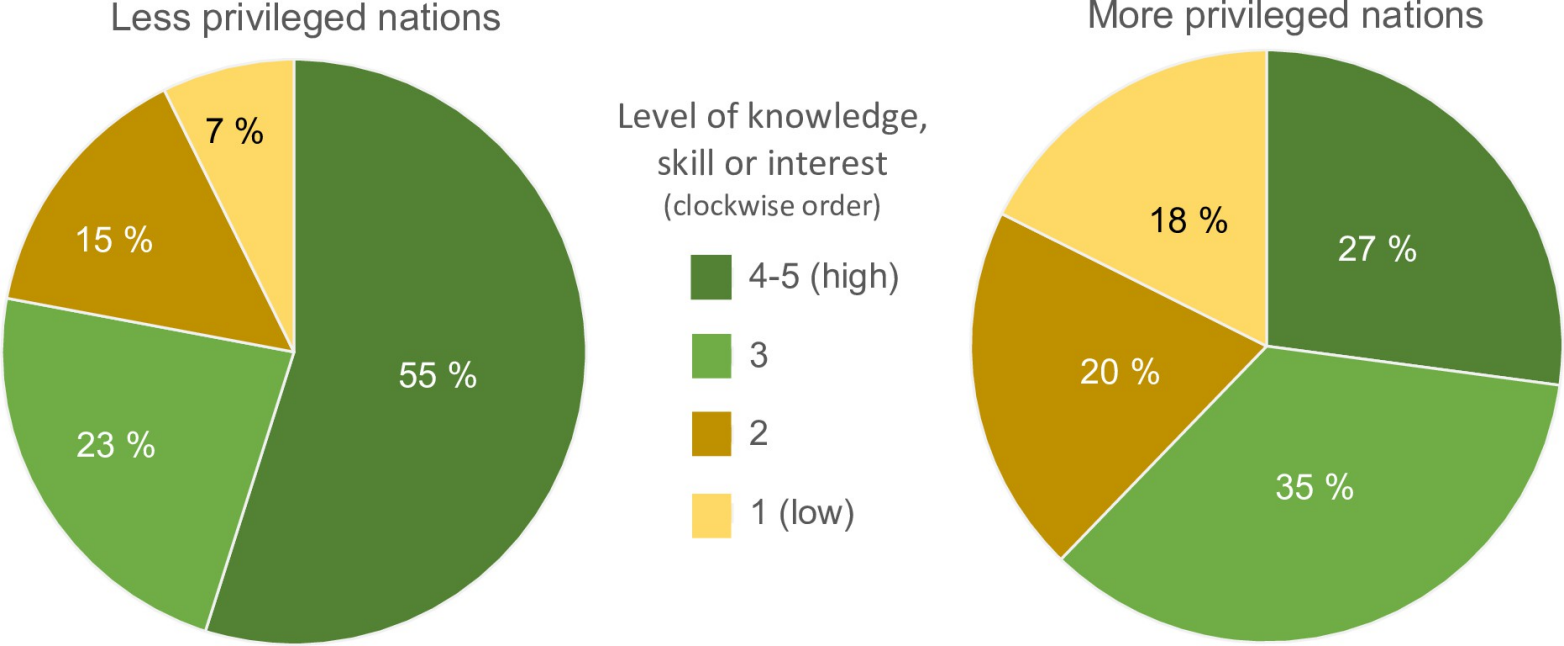
Teachers’ evaluation of pupils in their class, regarding levels of skills, knowledge and interests targeted in the open online course EDU-ARCTIC (2016–2019). N = 255 teachers with 25,398‬ pupils in the pre-survey, and 72 teachers with 3,428 pupils in the post-survey (survey types are merged in figure). Teachers were 76% females, and 99% from Europe or nations bordering Europe. We defined ‘less privileged’ as nations below median United Nations in-equality adjusted Human Development Index for Europe (IHDI = 0.815). The sample sizes for less and more privileged were 303 and 24 teachers, respectively.

In their evaluation, teachers from less privileged nations rated their pupils higher than did teachers from more privileged countries (Fig 5). The scores in the pre-survey were negatively related to the IHDI listing in all but 5 questions (test of average across all questions: Pearson’s = -26.1, df = 253, p<0.001). Because almost all respondents in the post-survey were from less privileged nations, we did not test for influence of IHDI on differences between pre- and post-surveys.

**Fig 6:**
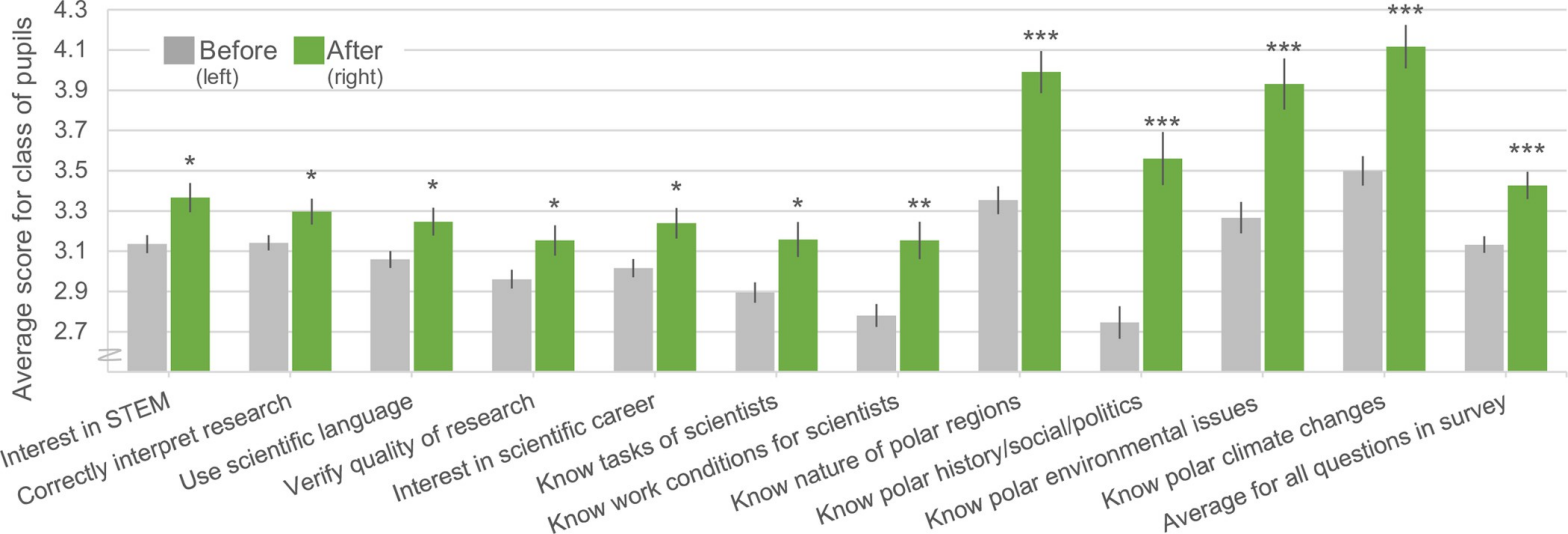
Teachers’ evaluation of pupils in their class (13–20 years of age), regarding levels of skills, knowledge and interests targeted in the open online course EDU-ARCTIC (2016–2019). N = 255 teachers with 25,398‬ pupils in the pre-survey, and 73 teachers with 3,428 pupils in the post-survey. Teachers were 76% females, and 99% from Europe or nations bordering Europe. Pupils were rated on a scale from 1 (low) to 4 (high). Note that the last four questions were rated on scale 1–5. Shown are all targets with a significant increase between the pre- and post-surveys (there were 3 additional targets without such increase). The ‘average for all questions in survey’ includes these plus 8 general learning skills not specified as targets in the project (i.e. all questions listed in Table 1 of this paper). Stars indicate significance of increase.

The teachers reported significant increases in all three categories of learning goals from the pre-survey to the post-survey (Table 1) (Fig 6). Averaged across all questions, the reported levels increased from 3.1 to 3.4 (t = 3.4, p≤0.001). The scores were the same when we looked at data from only teachers who participated in both surveys (N = 65), from 3.1 to 3.4 (paired test of increase: t = 4.7, p ≤0.001). Obviously, the mean pre-survey score for these teachers (3.1 ± SD 0.50) was not significantly different than the mean pre-survey score for the whole sample of respondents (3.1 ± SD 0.66) (t = -0.4, p = 0.659). The teachers reported that the pupils especially gained in their basic knowledge of polar areas (its nature, history, social specificities and politics), and in their knowledge of current environmental issues related to these regions. In contrast, there were no significant increase in the reported pupils’ *general* skills of learning, but these were not stated as specific learning goals of EDU-ARCTIC.

**Table 1:** Results of teacher-evaluations (online surveys) of learning outcomes for pupils 13–20 years of age participating in the open online course EDU-ARCTIC (2016–2019). Results pertain to the teacher’s collective assessment of the pupils in their class. N = 255 (46% of all active) teachers with 25,398‬ pupils in the pre-survey, and 73 (13%) teachers with 3,428 pupils in the post-survey. Scores (means ±1 SE) denote levels of skill/learning goal among the pupils, on scales 1–4 or 1–5, where 1 = lowest and 4 or 5 = highest. Questions marked with ^§^ comprise merged questions that asked for similar information, after testing each merged question separately and finding that they yielded similar results. Bolded questions = significant difference between pre- and post-survey.

Q#	Question text (shortened, see S1 Table in S1 File for complete text)	Pre-survey	Post-survey	Test of difference
*General skills (not stated learning goals of EDU-ARCTIC) (scale 1–4)*				
1_1_1	Use acquired knowledge in practice?	3.1 ± 0.09	3.1 ± 0.08	t = 0.4, p = 0.675
1_2_1^§^	Integrate knowledge across STEM fields and across external fields?	3.1 ± 0.08	3.2 ± 0.07	t = 1.3, p = 0.181
1_4_1	Involving themselves in experiments in class?	3.4 ± 0.07	3.4 ± 0.07	t = 0.6, p = 0.575
1_4_2	Independently design experiments?	2.9 ± 0.11	3.0 ± 0.09	t = 1.2, p = 0.250
1_5_1	Can logically conclude?	3.2 ± 0.08	3.3 ± 0.07	t = 1.2, p = 0.235
1_6_1^§^	Can realize tasks needed and engage in tasks in group?	3.4 ± 0.07	3.5 ± 0.06	t = 0.9, p = 0.367
1_7_1	Willingly use technologies to learn?	3.6 ± 0.07	3.6 ± 0.06	t = 0.5 p = 0.589
1_7_2	Have more effective learning due to technology?	3.5 ± 0.07	3.6 ± 0.07	t = 0.8, p = 0.452
*Learning goal (1) Enhanced knowledge about nature in polar areas and its global role in environmental issues (scale 1 to 5)*				
1_1_2	Interested in Polar/Arctic issues? (scale 1–4)	3.1 ± 0.09	3.2 ± 0.08	t = 1.5, p = 0.137
**3_1_1** ^**§**^	**About nature, geography and natural resources of polar regions**	**3.4 ± 0.14**	**4.0 ± 0.12**	t = 4.5, p≤ 0.001***
**3_1_5** ^**§**^	**About history, social and political specificities in polar regions**	**2.7 ± 0.17**	**3.6 ± 0.15**	t = 4.9, p≤ 0.001***
**3_1_6**	**About sensitivity to environmental issues in polar regions**	**3.3 ± 0.16**	**3.9 ± 0.14**	t = 4.1, p≤ 0.001***
**3_1_7**	**About climate change of polar regions**	**3.6 ± 0.15**	**4.1 ± 0.13**	t = 4.1, p≤ 0.001***
*Learning goal (2) Enhanced understanding of research and scientific language in environmental sciences (scale 1–4)*				
**1_3_1**	**Correctly interpret results of research?**	**3.1 ± 0.08**	**3.3 ± 0.07**	t = 2.0, p = 0.051*
**1_3_2**	**Able to use scientific language?**	**3.1 ± 0.09**	**3.2 ± 0.08**	t = 2.2, p = 0.032*
2_1_1	Formulate and justify research questions and hypotheses?	3.0 ± 0.09	3.1 ± 0.08	t = 1.2, p = 0.224
2_1_2	Apply adequate sources, tools and methods to test hypotheses?	2.9 ± 0.10	3.1 ± 0.09	t = 1.6, p = 0.120
**2_1_3**	**Verify the quality of research results?**	**3.0 ± 0.10**	**3.2 ± 0.09**	t = 2.0, p = 0.051*
*Learning goal (3) Familiarization with scientific career opportunities*, *and increased interest in pursuing such (scale 1–4)*				
**2_2**	**Showing interest in scientific careers?**	**3.0 ± 0.09**	**3.2 ± 0.08**	t = 2.4, p = 0.019*
**2_3**	**Showing interest in STEM?**	**3.1 ± 0.09**	**3.4 ± 0.08**	t = 2.5, p = 0.013*
**2_4**	**Knowledge about the vocational tasks of a professional scientist?**	**2.9 ± 0.11**	**3.2 ± 0.09**	t = 2.5, p = 0.015*
**2_5**	**Know the work conditions of scientists (salary, degree requirements)?**	**2.8 ± 0.12**	**3.2 ± 0.10**	t = 3.1 p = 0.002**

We also tested the reported learning outcomes separately for respondents from less privileged nations, in case the changes were an artefact of nationality bias in respondents between the pre- and post-surveys. The reported increases in learning outcomes were still highly significant, and of at least similar extent as for the whole sample of respondents. Averaged across all questions, the reported levels of pupils in less privileged nations increased from 3.1 to 3.4 (t = 3.4, p≤0.001).

## 4. Discussion

In this study, we analyzed participant demographics and teacher-evaluation of pupils’ learning outcomes in an informal open online course, to evaluate if it was used by less privileged learners, and to test if the teachers found that their pupils achieved three specific learning goals. Our results suggest that the course was efficient in both regards. We also gained unforeseen insights as to how future online course can be even more successful. We will discuss the above aspects separately, because they are not interdependent in our context of study.

### 4.1. Reaching the less privileged

We largely succeeded in reaching the less privileged (to democratize learning, as per **Challenge 1**), by both our criteria: (1) the UN human development index (IHDI), and (2) the urban-rural divide. The share of rural participants in EDU-ARCTIC likely was even an underestimate, because our application form asked for ‘name of town’ as their localization. Some of the most rural teachers, in lieu of not being located in a town, likely reported their location to a larger town nearby. As outlined in the introduction, open online courses (at least MOOCs for higher education) appear to generally attract already privileged learners [reviewed by 14, 39], and some conclude that these courses are not contributing to democratize learning [e.g., 9]. Our case study thus appears more of an exception than the norm in the scholarly literature on outcomes of open online course and the less privileged.

We identify the following four factors in our study, which likely contributed to the high participation of less privileged participants. While definitions of less privileged are case-specific, the four factors reveal a take-home-message that likely applies irrespectively of case. Which is: know your target group, and make deliberate efforts to recruit it. If the group normally is underrepresented in the type of online course you are providing, it will not self-recruit by passive means.

#### (1) The saturation factor

There likely is a greater level of saturation of access to open online courses in more privileged than in less privileged areas of Europe. The H2020 program of the European Union has funded vast amounts of money to education, including open schooling, and the money has mostly gone to beneficiaries in more privileged nations. After two years of operation, none of its top-50 beneficiaries within education were based in EU-13 countries [40], which essentially comprise nations with below-median IHDI listing, like Poland and Romania. Only 9 out of totally 600 top-50 placings across beneficiary categories (education, research, SMEs etc.) were beneficiaries from EU-13. One may therefore suggest that our high share of less privileged teachers may rather reflect a lack of interest from more privileged teachers. However, looking at the absolute number of teachers, we believe our case even shows a large interest from the less privileged. If this is a general pattern, open online courses should have higher rather than lower potential to reach less versus more privileged areas of the world.

#### (2) The effort factor

The EDU-ARCTIC organizing team was compiled to have members with established contacts specifically in nations with lower IHDI (Eastern Europe), and in rural areas irrespectively of IHDI. Extensive efforts were made to reach our target groups in these areas, as outlined in the method section. Overall, we spent approximately 9,000 working hours on recruiting teachers, directly in person or indirectly through media dissemination. We also participated on 182 smaller and larger educational events in 33 countries to present the project, both in-situ and online. The consortium coordinator was a national contact for SCIENTIX, an established network of science education in Europe with ambassadors in 27 countries. The efforts likely created a cascade effect rippling down from the stakeholders we were in direct contact with to their local individual networks.

#### (3) The language factor

We provided multi-linguistic learning components. This likely encouraged teachers with poorer English skills, who should coincide with our target group of less privileged. For example, only about 1/3 of Polish and Romanian people report to be able to hold a conversation in English [41]. Data from students seeking to improve their English skills likewise suggests a positive correlation between HDI and their English proficiency [42, p. 35]. We observed English language barriers during our project too. More pupils (and teachers) were vocally active during the webinars in national languages compared to in the English lessons. While we sought to actively engage the participants (see method section 2.2), and similarly so in any language version of the webinars, we largely failed to have pupils being active in the English versions of our webinars. On the other hand, we did not notice higher activity of pupils from nations with higher English proficiency. However, the share of high IHDI pupils were too few to conclude.

#### (4) The competition factor

We motivated and monitored activity of teachers by assigning each with an accumulating activity score (“Edu-Game”). A public list of top scorers was continuously updated in our social media. Top scorers received special awards like exclusive online lessons for their class and diplomas. This appeared more motiving for teachers in the less privileged nations, especially in certain nations with the lowest IHDI. Some research suggests a negative correlation between HDI and endorsement of competition [e.g., 43]. Competitiveness may be a culturally inherited motivation by itself, but it may also correlate with differences in current working conditions. For example, less secure employment contracts, and higher needs to document excellence to obtain higher salaries. These are components of economic freedom, which generally are lower in nations low on HDI (ibid.). If an open online course targets such nations, it may therefore be more beneficial to include an element of competition.

Importantly, the group we reached the least was rural males in less privileged nations. If a rural male registered for the EDU-ARCTIC course, he was more likely than rural females to be from a more privileged nation. We have not been able to retrieve data on rurality linked to gender ratios of European teachers, but there is a general bias towards female teachers in the secondary schools [64%, 44], and this bias is stronger in less privileged nations (e.g., 70% in Poland and Romania, ibid). It may also be that rural male teachers are particularly less inclined to seek extracurricular activities. A series of studies suggest that male teachers in general may be so inclined, because of differences between male and female teaching styles. Females, compared to males, spend a smaller portion of class time lecturing and a greater portion of class time on active practices [45], are more engaged [46], and more informal and interactive toward students’ ideas [47]. These differences likely make female teachers more likely to search for and use extracurricular resources. Notably, two of these three studies are from higher education, and it may be that males teach more like females at lower age levels. If so, this is not one of the reasons why we reached less of the rural male teachers with our project. Also notably; once becoming active in the EDU-ARCTIC, there were no strong and consistent gender or rurality differences in activity levels (Figs 3B and 4).

### 4.2. Monitoring of learning

The teachers reported significant increases for all the three aspired learning outcomes for their pupils. They reported that the pupils especially gained in their basic knowledge of polar areas (its nature, history, social specificities and politics), and in their knowledge of current environmental issues related to these regions (**learning goal #1**). Likely contributing to these reported learning outcomes was the fact that polar issues currently is a hot topic across global societies, not the least among young people. Starting around the same time as the onset of our project period, young people globally have showed unusual engagement in climate change [sensu Greta Thunberg’s “skolstrejk for klimatet”, 48]. Social media with hashtags on climate change increased manifold from 2008 to 2018 [49], and press news about polar regions is frequently linked to these posts, through environmental (and often controversial) issues with for example polar bears [50] and ice melting [51]. Therefore, the pupils in our case study likely was doubly motivated to learn about the topic by having both peer interest and a particularly engaged teacher (see below).

Secondly, the teachers reported that their pupils gained significant learning about how it is to work as a scientist (**learning goal #3**). Most importantly, the teachers evaluated the pupils to have a greater interest in pursuing a STEM careers themselves, as a consequence of participating in the EDU-ARCTIC open online course. Because they also reported that their pupils gained in their understanding and application of scientific language (for critical thinking, as per **learning goal #2**), the course may also have provided the young with socially practicable, not only content-based, scientific literacy. The European Union emphasizes that science education is vital to develop a culture of responsible thinking and evidence-based reasoning for sustainable decision making [52]. This demands scientific literacy in a broad sense, where the society achieves “*education through science*, *as opposed to [only] science through education*” [53, p. 275]. As outlined in our introduction, we believe the blended learning environments created by open online courses like ours may be particularly suited for this, especially the informal ones. It brings youth in direct contact with scientists and research. This is something that learners normally will not have access to until they start graduate studies, which means large segments of youth never will experience it.

There exists no objective way to evaluate learning outcomes. Evaluations are coloured by a range of individual and contextual factors, not the least how survey questions are formulated [54]. Teachers who do collective class-evaluations may be biased by, for example, the presence of a few particularly vocal or cognitively intelligent pupils [55], or pupils that they feel more or less similarity with [56]. However, this bias should be similar in the pre-survey as in the post-survey, assuming that our teachers in general were already well acquainted with their pupils when they joined the project. One aspect of our approach that reduced the influence of these factors, is that we used the exact same questions in our pre-survey as in our post-survey. If, for example, a teacher scored the class higher due to being biased by a few pupils, the teacher would score the class likewise higher in both surveys. When evaluating the learning, we looked at relative change in the Likert scores. The absolute values of the scores did not matter, and as such had no mathematical bearing on our results.

Another potential bias is teacher motivation and self-efficacy. There were no obligatory activities in EDU-ARCTIC, and expectedly, there were differences in which teachers completed the pre- and the post-surveys. Based on, e.g. the activity scores, it is likely that the more dedicated teachers were over-represented among the post-survey teachers. Our project was also organized specifically to provide teachers with a learning scenario using extracurricular activities and possibly unfamiliar technologies, which requires more personal effort and self-efficacy from the teachers [57, 58], than does a fixed curricula where one lectures about familiar topics in familiar manners. A more engaged teacher may be more committed to being “a good respondent” in interviews about their (pupils’) performance (social desirability bias [59]).They could therefore, deliberately or unconsciously, have rated their pupils higher in the post-survey than in the pre-survey simply for this reason. However, we did not provide teachers with the scores they had given during the pre-survey. Unless they had made efforts to personally archive their scores (a big task to do manually, as there were 26 questions with 4 or 5 categories, split for boys and girls), they participated in the post-survey not knowing how they had scored their pupils in the pre-survey. Our data also support that the presumably more engaged teachers showed no “good respondent” bias, because the average score across questions in the pre-survey was the same for those participating in both surveys as it was for the whole sample of respondents.

Given that the teachers in our surveys may have been more engaged than typical, this also coloured the sample of pupils in the surveys. For example, these pupils likely received more than typical individual attention and encouragement from their teachers to engage in interactive learning. This bias does not take away from the positive outcomes of our project. It only cautions us how we interpret the generality of our results. Our course proved a valuable extra-curricular resource for *engaged* teachers to increase learning outcomes for their pupils. Possibly, some of these teachers became engaged from following the course, but it may also be that they were more engaged to begin with.

### 4.3. Unexpected lessons learnt

We found that many teachers used the project for their own education, not only as a tool for teaching pupils. This was particularly evident in teachers showing up for the webinars without a class of pupils. The EDU-ARCTIC course therefore served also as continued education for teachers. Many educators have little sense of self-efficacy when it comes to the socio-ecologically tangled web of environmental issues [e.g., 60]. This may of course not be limited to environmental issues. The PISA survey 2018 found that “*fewer than 1 in 10 students in OECD countries was able to distinguish between fact and opinion*” [61, p. 14]. This is partly a result of teachers struggling with the same. Many teachers have to cover a wide range of the school curricula and cannot have up-to-date expertise in all of them. Again, the ‘informality’ of our course may be a key here. There were no obligations involved for the teachers, yet they had full access to all learning components. They could initiate direct contact with a specialist scientist and discuss complex issues. A high share of teachers likely experience impoverished networks that do not offer sufficient contact with within-subject-specialist teachers [e.g., 62]. Enrolling for *formal* continued education is generally also perceived to be highly constrained by time and money (ibid.).

## 5. Conclusions and advice for future studies

Based on our case-study, we conclude that informal and open online courses can be of particular interest to less privileged learners, and supposedly, in time, contribute to democratize learning. Furthermore, they can contribute to increase sustainable citizenship, most importantly by bringing both youth and educators in direct contact with specialist scientists. As indicated by the teacher-evaluations, the course contributed to improve the pupils’ scientific literacy, both content-wise and in a socially practicable sense. In order to give data on learning outcomes credibility, we emphasize that the monitoring of learning and its sources of bias, must be carefully considered ahead of starting the course. This holds, of course, to any type of educational courses, formal or informal.

We believe informal STEM based courses like ours facilitate education through science rather than science through education, which is much needed for sustainability in a world where not everybody can or should pursue academic careers. The EDU-ARCTIC network of more than 1800 teachers and educators (as per March 2021) gave opportunity to further develop these lines of activities, and are being utilized in a follow-up EEA funded new project *EDU-ARCTIC 2*. We also want to emphasize that recruiting our target groups and keeping participants active required deliberate and extensive efforts. Our take-home message to future providers of similar courses is that one should not assume that participants, will self-recruit by passive means, especially the less privileged who may be less motivated to begin with.

## Supporting information

S1 File (PDF)

S1 Dataset (XLSX)
