# Supplementary material for: Democratizing education: Open schooling engaged the less privileged in environmental sciences
Source: PLoS One. 2022 Apr 8;17(4):e0266655. doi: 10.1371/journal.pone.0266655 (PMC8992980; doi:10.1371/journal.pone.0266655)
Supplement: S1 File — (PDF) [file pone.0266655.s001.pdf]

S1 Table 1. List of unique topics for online lessons given in the open schooling project EDU-ARCTIC 2016-2019. Several topics were given in multiple languages, and/or given multiple times. A topic is listed only once (in the main language it was given). The topics are listed in alphabetical order.

	Title of lesson	Times
(1)	… σσς, η Γη είναι Ζωντανή! (EN: …ssshhh, Earth is alive!)	1
(2)	100 questions to a polar researcher	2
(3)	130.000 years of climate change in Borðoyavík, Faroe Islands	5
(4)	7 wonders of the Nature	2
(5)	A Snowball Earth: how, what, why and when?	1
(6)	A story of maps	1
(7)	A virtual walk through a DNA laboratory	1
(8)	Adapt or die: how do organisms react to climate change	5
(9)	Adaptations in Arctic mammals to a life on the edge	2
(10)	All the colours of the Aurora	4
(11)	All year outdoor activities with kids in the Arctic: the art of variation	1
(12)	Ancient Eskimo Societies	2
(13)	Aquaponics í Føroyum (EN: Aquaponics in the Faroe Islands)	1
(14)	Arctic and Antarctic fauna - comparison	3
(15)	Arctic climate and environment – what are the challenges we face?	1
(16)	Arctic hydrology: Where does the water go?	3
(17)	Arctic rivers	3
(18)	Arctic tundra - adaptations of plants to the climate	4
(19)	Aurora - The lights of the dark sky	5
(20)	Bears, bear poop and DNA	3
(21)	Belgian Antarctic Expedition	5
(22)	Bezpieczeństwo w Arktyce (EN: Safety in the Arctic)	1
(23)	Biological clocks in the Arctic	4
(24)	Birth and death of an ocean	4
(25)	Can geotourism and geoparks contribute to nature conservation in the North Atlantic region?	1
(26)	Citizen monitoring: how to participate in monitoring system	1
(27)	Climate change	1
(28)	Climate change: myths vs. science	5
(29)	Climate modelling: key to our past and future	3
(30)	Co nas czeka - zlodowacenie czy potop? (EN: What will the future bring-glaciation or flood?)	2
(31)	Comprendre le tourbillon stratosphérique arctique en hiver	1
(32)	Contemporary Inuit Societies	1
(33)	Day vs Night in the Arctic	3
(34)	Designing a weather forecast	1
(35)	Digital maps and geographical coordinates – for upper secondary schools	1
(36)	Diving across the animal kingdom – when the search for food drives the evolution	2
(37)	Do brown bears believe in gender stereotypes?	2
(38)	Do you know what minerals are used for?	3
(39)	Drifting ice stations	4
(40)	Dzień Morza (EN: Sea day)	1
(41)	Dzień Polarnika (EN: Polar Day)	2
(42)	Earth as a jigsaw - plate tectonics in Europe	1
(43)	Earth from Space - global changes	1
(44)	Earthquakes	1
(45)	Earthquakes - not only colliding continents	1
(46)	Earth's as a jigsaw - litosphere and plate tectonics	3

(47)	Earth's history in speed up	4
(48)	Earth's history in speed up - life on Earth	3
(49)	Earth's magnetic field	4
(50)	EDU-ARCTIC Competition	2
(51)	EDU-ARCTIC Competition 2018: get inspired!	1
(52)	EDU-ARCTIC Monitoring System - introductory	1
(53)	Epoki lodowcowe (EN: Ice ages)	1
(54)	Everything you wanted to know about maps, but were afraid to ask	2
(55)	Fabulous freshwater pearl mussels - the speakers of clean rivers	1
(56)	Field equipment of scientific investigations	1
(57)	Fizyka trzęsień ziemi (EN: Physics of earthquakes)	1
(58)	Flight of the swans	1
(59)	Flying penguins	6
(60)	Freeze tolerance: why do not animals become ice during winter?	1
(61)	From Antarctica: Which species of mammals you can see on King George Island	1
(62)	Frozen ground	3
(63)	Geoengineering: damnation or salvation?	3
(64)	Geografia obszarów okołobiegunowych 1. (EN: Geography of circumpolar areas 1)	2
(65)	Geografia obszarów okołobiegunowych 2. (EN: Geography of circumpolar areas 2)	2
(66)	Geological field observations – what is oldest?	2
(67)	Geothermal energy	1
(68)	Glaciation: to keep to survive	2
(69)	Glacier processes and landforms	1
(70)	Glaciers	1
(71)	Glaciers and glaciations: introduction	1
(72)	Glaciospeleology: hobby, sport, or science?	4
(73)	Great polar expeditions	3
(74)	Greenland: Kalaallit Nunaat in a nutshell	10
(75)	How Arctic indigenous peoples observe changes in climate and biodiversity?	2
(76)	How can ice cover of the Arctic Ocean influence on weather in Europe?	2
(77)	How can people live among the rocks?	4
(78)	How can the Arctic save us from global catastrophe?	1
(79)	How glaciers shape the Earth's surface?	1
(80)	How it's made: Satelite photos	12
(81)	How the Arctic charr cope with everything	2
(82)	How to become a polar researcher?	1
(83)	How to pluck a brown bear	1
(84)	Hvar er Norðpólurin? (EN: Where is the North Pole?)	1
(85)	Hvordan kan vi bruke EDU-ARCTIC I den norske skolen? (EN: How can we apply EDU-ARCTIC at Norwegian schools?)	1
(86)	Hydropower	1
(87)	Ice age in the Faroe Islands	3
(88)	Iceland: The land of air and water	7
(89)	Identifying cloud types	4
(90)	Imperial Transantarctic Expedition - Ernest Shackleton	4
(91)	Inside Polish Polar Station	1
(92)	Insights after 10 years of genetic monitoring of brown bears	1
(93)	Introductory webinar for new Albanian teachers	1
(94)	Introductory webinar for new teachers	5
(95)	Invatam. Experimentam.Ne protejam....Seismologia pe intelesul elevilor din ciclul gimnazial (EN: Learn. Experiment.Protect.....Seismology for secondary school)	1
(96)	Is it time to build an Ark? Sea level rise.	3
(97)	Jak zostać polarnikiem? (EN: How to become a polar explorer)	1
(98)	Jørðin og Føroyar í jarðfrøðiligari tíð	1
(99)	Książki o Dalekiej Północy	1
(100)	LiDAR: Laser in humanity's service	10

(101) Life and work at the Polish Polar Station Hornsund on Svalbard	7
(102) Living in the Arctic – different aspects of life in extreme environment	1
(103) Living in the Polish Polar Station – technical aspects	2
(104) LODOWCE - cz. 1 (EN: Glaciers - Part 1)	1
(105) LODOWCE - cz. 2 (EN: Glaciers - Part 2)	1
(106) Lodowce i zlodowacenia - część 2 - cykl "Wyzwanie dla klas"	1
(107) Lodowce i zlodowacenia - część 3 - cykl "Wyzwanie dla klas"	1
(108) Lodowce i zlodowacenia - część 4 - cykl "Wyzwanie dla klas"	1
(109) Lodowce i zlodowacenia - część 1 - cykl "Wyzwanie dla klas"	1
(110) Lodowce i zlodowacenia (wstęp)	1
(111) Lodowce i zlodowacenie cz. 3. Procesy towarzyszące lodowcom i lądolodom (EN: Glaciers and glaciation, part 3. Processes associated with glaciers and ice sheets)	1
(112) Looking into the Earth	2
(113) Mass balance of glaciers	6
(114) Measuring equipment in Svalbard	1
(115) Megathrust Earthquakes	3
(116) Mercury and lead pollution in the Arctic – where does it come from?	3
(117) Meteorological phenomena and measurements on Spitsbergen	2
(118) Microbial factories: great power of tiny organisms	2
(119) Mikroby w Arktyce - sekretne życie lodowców (EN: Microbes in the Arctic - secret life of glaciers)	1
(120) MIMOSA	1
(121) Monitoring System Mobile App	2
(122) Mushrooms in nature – our helpers	3
(123) Niesporczaki: sztuka przetrwania	2
(124) Northern lights - a magic spectacle in the sky	3
(125) Northern seas, why is all the fish there?	2
(126) Nurkowanie swobodne: hobby, sport, nauka (EN: Free diving: hobby, sport, science)	1
(127) Ocean currents: the dance of water	3
(128) On the top of Atlantic Rift	3
(129) Our life in CHAOS: butterfly effect and science	3
(130) Paleomagnetism - the story of Earth	3
(131) Palsas – first stages of permafrost	1
(132) Paradoksy matematyki (EN: Paradox of mathematics)	1
(133) People in the Arctic - Introduction	1
(134) Permafrost and periglacial processes	1
(135) Phenology part 1: The rhythm of the flowers and animals through the year	3
(136) Phenology part 2: The plants in EDU-ARCTIC Monitoring System	3
(137) Phenology part 3: The insects in EDU-ARCTIC Monitoring System	3
(138) Phenology part 4: The birds in EDU-ARCTIC Monitoring System	3
(139) Plastic in the Arctic – it's in the air and oil too!	1
(140) Plastic in the Arctic - alarming consequences for the wildlife	2
(141) Pleistocene MEGAFAUNA	5
(142) Polacy na biegunach (EN: Poles on poles:))	1
(143) Polar bear - the king of Arctic	6
(144) Polar bear vs. penguin	4
(145) Polar Explorer Day	2
(146) Polar lows	3
(147) Polar night and midnight sun - one year in the Arctic	2
(148) Polar Psychology	4
(149) Polar regions: laboratory of space exploration	4
(150) Polarne aspekty medyczne (EN: Medical aspects in Polar regions)	1
(151) Polish Polar Station in Hornsund	5
(152) Polska Stacja Polarna od środka	1
(153) Pomiary meteorologiczne w Arktyce (EN: Meteorological observations in the Arctic)	2
(154) Procesy i formy glacjalne (EN: Glacial processes and forms)	1

(155) Procesy i zjawiska peryglacjalne (EN: Periglacial processes and phenomena)	1
(156) Prosto z Antarktyki: Życie i praca w Polskiej Stacji Antarktycznej – kim jesteśmy, co robimy, gdzie mieszkamy? (EN: Directly from Antarctica: work and life in Polish Antarctic Station)	1
(157) Przejście Północno – Zachodnie – historia eksploracji (EN: Northwest Passage - exploration history)	1
(158) Ptarmigan hunting in northern Norway	1
(159) Reindeer, caribou, hreindyri: the dearest deers	3
(160) Renewable energy	1
(161) Research activity at the Stanisław Siedlecki Polish Polar Station in Hornsund	1
(162) Research station NIBIO Svanhovd: living and doing research in the far north	2
(163) Ruch lodowców (ćwiczenia praktyczne) (EN: Glaciers, practical exercies)	1
(164) Rzeźbotwórcza działalność lodowców górskich i lądolodów (EN: Landshaping processes of mountain glaciers and ice sheets)	2
(165) Saami: the Reindeer People	8
(166) Safety in Svalbard	2
(167) Schimbăm date - localizăm cutremure (EN: We exchange data - we locate earthquakes)	2
(168) Scientific hypotheses – the backbone of science	1
(169) Scientix workshop 1	2
(170) Scientix workshop 2	2
(171) Settlements in Svalbard	4
(172) Sheep in the Land of Fire and Ice Q&A	2
(173) Siberian people	2
(174) Sixth Mass Extinction	3
(175) Skąd się bierze woda w Arktyce? (EN: Where does Arctic water come from?)	1
(176) Snow chickens	2
(177) Space weather: what, why, how, when	5
(178) Spotkanie informacyjne dla nauczycieli (EN: Information webinar for teachers)	1
(179) Spotkanie z Anną Nadolną (EN: Meet a researcher Anna Nadolna)	1
(180) Spotkanie z Dagmarą Bożek (EN: Meet a researcher Dagmara Bozek)	3
(181) Strefy klimatyczne kuli ziemskiej (EN: Climate zones on Earth)	1
(182) Sustainable energy	1
(183) Tajemnicze wnętrza lodowców (EN: Mistery of glacier interior)	3
(184) Tardigrades - the ultimate survivors	4
(185) Teacher workshop	1
(186) Teacher workshop - only for participants of workshop in Bucarest	1
(187) Teacher workshop - only for participants of workshop in Oslo	1
(188) Technology for ships and vessels	2
(189) The Andes	1
(190) The Arctic, people's land	12
(191) The bears are back! End of winter sleep	1
(192) The biggest mysteries that science can't solve (yet)	2
(193) The Earth and the Faroese Islands in geological time	1
(194) The Great Migrations	3
(195) The greatest trade route that the world has never heard about: The Tea Horse Road	1
(196) The greylag goose – a goose “too well” adapted to its environment?	2
(197) The mystery of glaciers’ interior	1
(198) The polar vortex	1
(199) The rock cycle	4
(200) The secret invasion: alien species	3
(201) The secret life of Arctic plankton	4
(202) The secrets of sub-surface: minerals and ores	1
(203) Tourism in polar regions	1
(204) Tourists in the polar station	5
(205) Tsunami risk in the North Atlantic Ocean	4
(206) Tvøst og spik sum mannaføði (EN: Grind as a food source)	1

(207) Ultraviolet radiation – is it always an enemy?	3
(208) Understanding the Arctic Stratospheric Vortex in Winter	1
(209) Understanding the concept of climate	2
(210) Unfantastic climate change and how to find it	6
(211) Venus	2
(212) Vikings: northern warriors	3
(213) Volcanoes and humans	8
(214) Vremea – un joc între presiune și temperatură – Liceu (EN: Weather - a game between pressure and temperature)	4
(215) Warsztaty EDU-ARCTIC - spotkanie z polarnikiem	1
(216) Water resources of the Arctic	2
(217) Weirdest Arctic animals	10
(218) Weirdest Arctic fun facts	5
(219) Whales - Arctic giants	3
(220) What happens in the Arctic, doesn't stay in the Arctic: climate change	4
(221) What is academic writing like?	3
(222) What is citizen science?	4
(223) What is DNA, and how can we use it in science?	1
(224) When living is toxic: bioaccumulation and biomagnification	3
(225) Where did it all come from? Origins of life	3
(226) Where is the North Pole?	2
(227) Why and how to study brown bears?	1
(228) Why do many trees have bright coloured leaves in autumn?	1
(229) Why the Arctic is a laboratory of contemporary international relations	1
(230) Wielkanoc na stacji polarnej (EN: Easter at polar station)	1
(231) Wielki dryf FRAMA (EN: FRAM. The great drift)	1
(232) Wieloletnia zmarzlina	1
(233) Wildlife forensic – using DNA to protect wildlife	1
(234) Woda - niezwykłe medium w przyrodzie (EN: Water - amazing medium in nature)	1
(235) Work and life in Polish Arctic and Antarctic research stations	4
(236) Wszystko, co chcielibyście wiedzieć o mapach, ale baliście się zapytać (EN: All you wanted to know about maps, but did not dare ask about)	2
(237) Wyprawa JEANNETTE (EN: JEANETTE expedition)	1
(238) Wyścig do bieguna południowego (EN: Race for the South Pole)	1
(239) Zasoby wodne Arktyki (EN: Water resources of the Arctic)	1
(240) Zlodowacenia górskie w Polsce (EN: Mountain glaciation in Poland)	2
(241) Zlodowacenia i rzeźba postglacjalna Polski - część 1	3
(242) Zlodowacenia i rzeźba postglacjalna Polski - część 2	1
(243) Zmiany zlodowacenia Arktyki, część 1 - lodowce (EN: Changes in the ice coverage in the Arctic - Part 1 - Glaciers)	4
(244) Zmiany zlodowacenia Arktyki, część 2 - Rozwój i ruch lodowców, zlodowacenia (EN: Changes in the ice coverage in the Arctic - Part 2)	4

S1 Table 2. Scores given for each participation in an activity in the open schooling project EDU-ARCTIC 2016-2019. Scores were given to both motivate and monitor participant activity. We published and continuously updated the list of top scorers (first name and nation) on the project portal, social media and newsletters. Top scoring teachers and schools received special awards (diplomas and exclusive online lessons for their class). The mean score achieved among active teachers (engaging in at least one activity) during the project period was 30 ± SD 108, while the median was only 2 (77% of participants had a score of <10, and 11% had a score >50. The max score achieved was 1285.

**Activity**	**Score**
Webinars (participation, evaluation)	40, 40
Arctic competition (submission, finalist, winning, evaluation)	50, 100, 200, 50
Educator workshop (participation, evaluation)	200, 40
Monitoring System (initial registration, per report)	50, 10
Polarpedia (per term translated to national language	20
Documented dissemination about the project (educational events and such)	150
Self-evaluation survey (reporting on learning outcomes)	200

S1 Table 3. Questions asked in online surveys for self-evaluation of learning in the open schooling project EDU-ARCTIC 2016-2019. Surveys were sent at the onset and end of the project to the participating teachers, who evaluated jointly all the pupils (13-20 years of age) in their class. The questions were asked in both surveys.

Qst. #	Phrasing of question and response alternatives, as given in the survey
1_1_1	Do your pupils use the acquired knowledge in practice? (4 – Definitely use it 3 – Tend to use it 2 – Tend not use it 1 – Definitely do not use it)
1_1_2	Are your pupils interested in issues related to the Arctic? (4 - Definitely interested 3 - Quite interested 2 - Not very interested 1 - Definitely not interested)
1_2_1	Do your pupils integrate knowledge from various fields of mathematics and natural sciences (e.g. they use information obtained on other subjects while participating in your lesson)? (4 - Definitely integrate 3 – Often integrate 2 - Rarely integrate 1 - Definitely do not integrate)
1_2_2	Do your pupils explain external phenomena (e.g. natural, social, etc.) using the concepts acquired during the act of learning? (4 - Definitely explain 3 - Often explain 2 - Rarely explain 1 - Definitely do not explain)
1_3_1	Do your pupils correctly interpret the results of experiments, results of research? (4 - Definitely yes 3 - Rather yes 2 - Rather no 1 - Definitely no)
1_3_2	Are your pupils able to use scientific language, which you use in a class (e.g. use the same terminology)? (4 - Definitely are able 3 - Rather are able 2 - Rather are not able 1 - Definitely are not able)
1_4_1	Are your pupils enthusiastically involved in research processes or experimental processes which you propose during your lesson? (4 - Definitely involved 3 - Rather involved 2 - Rather are not involved 1 - Definitely are not involved)
1_4_2	Do your pupils independently design the experimental, research process? (4 - Definitely design 3 - Rather design 2 - Rather do not design 1 - Definitely do not design)
1_5_1	Can your pupils logically conclude? (4 - Definitely can 3 - Rather can 2 - Rather cannot 1 - Definitely cannot)
1_6_1	Can your pupils realize tasks within group? (4 - Definitely can 3 - Tend to be able to 2 - Tend not to be able to 1 - Definitely cannot)
1_6_2	Are your pupils willingly engaged in various of tasks within group? (4 - Definitely engaged 3 - Tend to be engaged 2 - Tend not to be engaged 1 - Definitely are not engaged)
1_7_1	Do your pupils willingly use modern technologies in order to learn? (4 - Definitely do so 3 – Tend to 2 - Tend not to 1 - Definitely do not use)
1_7_2	Do you think modern technologies have an impact on raising the effectiveness of learning process among your pupils? select the most appropriate. (4 – Yes, definitely 3 – Quite 2 - Not very much 1 - Definitely no)

2_1_1	Knowledge about formulating research questions and hypothesis (Can your pupils formulate questions? Can your pupils formulate objectives of research? Can your pupils justify formulated objectives of research?) (4 - very efficiently 3 – rather efficiently 2 - quite incapable 1 – definitely incapable)
2_1_2	Knowledge about applying adequate tools and methods to test the hypothesis (Are your pupils familiar with the scientific method of verification in the area of STEM? Do your pupils know examples of research in the area of STEM? Do your pupils have a knowledge about searching for reliable sources of information about scientific method and tools? Can they use these scientific method and tools effectively?) (4 - very efficiently 3 – rather efficiently 2 - quite incapable 1 – definitely incapable)
2_1_3	Can your pupils verify the quality of research results? (Whether the purpose, objective of the research was achieved, whether there is a need to another attempt, whether the resulting data are inconclusive or ambiguous?) (4 - very efficiently 3 – rather efficiently 2 - quite incapable 1 – definitely incapable)
2_2	Are your pupils showing interest in scientific careers? (4 - Definitely are showing 3 - Rather are showing 2 - Rather are not showing 1 - Definitely are not showing)
2_3	Are your pupils showing interest in STEM? (4 - Definitely are showing 3 - Rather are showing 2 - Rather are not showing 1 - Definitely are not showing)
2_4	Have of your pupils got a knowledge about the vocational tasks of a professional scientist? (4 - Definitely are showing 3 - Rather are showing 2 - Rather are not showing 1 - Definitely are not showing)
2_5	Do your pupils know anything about the conditions of work of professional scientists (e.g. possibilities of employment, salary, requirements to obtain a degree)? (4 - Definitely are showing 3 - Rather are showing 2 - Rather are not showing 1 - Definitely are not showing)
3_1_1	Knowledge about nature of polar regions (5 - very good 4 – quite good 3 – average 2 – not very good)
3_1_2	Knowledge about geography of polar regions (5 - very good 4 – quite good 3 – average 2 –not very good)
3_1_3	Knowledge about natural resources of polar regions (5 - very good 4 – quite good 3 – average 2 –not very good)
3_1_4	Knowledge about history of polar regions (5 - very good 4 – quite good 3 – average 2 – not very good)
3_1_5	Knowledge about social and political specificities concerning polar regions (5 - very good 4 – quite good 3 – average 2 –not very good)
3_1_6	Knowledge about sensitivity to environmental issues concerning polar regions (5 - very good 4 – quite good 3 – average 2 –not very good)
